# Pharmacokinetics of Intranasally Administered Dexmedetomidine in Chinese Children

**DOI:** 10.3389/fphar.2019.00756

**Published:** 2019-07-05

**Authors:** Cheng-Yu Wang, Harald Ihmsen, Zhi-Yan Hu, Jia Chen, Xue-Fei Ye, Fang Chen, Yi Lu, Jürgen Schüttler, Qing-Quan Lian, Hua-Cheng Liu

**Affiliations:** ^1^Department of Anesthesiology, Perioperative and Pain Medicine, The Second Affiliated Hospital and Yuying Children’s Hospital of Wenzhou Medical University, Zhejiang, China; ^2^Key Laboratory of Anesthesiology of Zhejiang Province, The Second Affiliated Hospital and Yuying Children’s Hospital of Wenzhou Medical University, Zhejiang, China; ^3^Department of Anesthesiology, Friedrich-Alexander-University Erlangen-Nürnberg (FAU), Erlangen, Germany

**Keywords:** dexmedetomidine, intranasal, pharmacokinetics, pediatrics, Chinese

## Abstract

**Background:** Intranasal application is a comfortable, effective, nearly non-invasive, and easy route of administration in children. To date, there is, however, only one pharmacokinetic study on intranasal dexmedetomidine in pediatric populations and none in Chinese children available. Therefore, this study aimed to characterize the pharmacokinetics of intranasally administered dexmedetomidine in Chinese children.

**Methods:** Thirteen children aged 4 to 10 years undergoing surgery received 1 µg/kg dexmedetomidine intranasally. Arterial blood samples were drawn at various time points until 180 min after dose. Dexmedetomidine plasma concentrations were measured with high performance liquid chromatography (HPLC) and mass spectrometry. Pharmacokinetic modeling was performed by population analysis using linear compartment models with first-order absorption.

**Results:** An average peak plasma concentration of 748 ± 30 pg/ml was achieved after 49.6 ± 7.2 min. The pharmacokinetics of dexmedetomidine was best described by a two-compartment model with first-order absorption and an allometric scaling with estimates standardized to 70-kg body weight. The population estimates (SE) per 70 kg bodyweight of the apparent pharmacokinetic parameters were clearance CL/F = 0.32 (0.02) L/min, central volume of distribution V1/F = 34.2 (4.9) L, intercompartmental clearance Q2/F = 10.0 (2.2) L/min, and peripheral volume of distribution V2/F = 34.9 (2.3) L. The estimated absorption rate constant was Ka = 0.038 (0.004) min^−1^.

**Conclusions:** When compared with studies in Caucasians, Chinese children showed a similar time to peak plasma concentration after intranasal administration, but the achieved plasma concentrations were about three times higher. Possible reasons are differences in age, ethnicity, and mode of administration.

## Introduction

Dexmedetomidine is a highly selective alpha2-adrenergic receptor agonist, which possesses sedative, anxiolytic, and analgesic properties (Belleville et al., 1992). Dexmedetomidine is characterized by a rapid and wide distribution throughout the body and a high-protein binding of about 94%, mainly to albumin and α1-glycoprotein (Weerink et al., 2017). Dexmedetomidine is eliminated mainly through biotransformation by the liver via direct N-glucuronidation by uridine 5′-diphospho-glucuronosyltransferase (UGT2B10, UGT1A4) and via hydroxylation mediated by cytochrome P450 enzymes, mainly CYP2A6 (Weerink et al., 2017). Although dexmedetomidine causes hypertension and bradycardia (Ebert et al., 2000), it has been shown that it can help to provide hemodynamic stability during stressful events in surgery (Kunisawa et al., 2011) and during induction of anesthesia (Yildiz et al., 2006). Furthermore, it has minimal effects on respiration and cognitive function (Belleville et al., 1992; Hsu et al., 2004). In pediatric anesthesia, dexmedetomidine is widely used and plays a significant role as an anesthetic adjuvant, in reducing postoperative pain, emergence agitation, and shivering (Mason and Lerman, 2011). Intranasal application is a nearly non-invasive and easy route of administration, which is comfortable and effective and does not require children’s cooperation (Jun et al., 2017). Intranasal administration of dexmedetomidine showed rapid absorption, rapid onset, and high bioavailability (Miller et al., 2018). To decrease side effects and optimize clinical dosing, it is important to get pharmacokinetic information on dexmedetomidine in pediatric patients. The pharmacokinetics of intravenous dexmedetomidine in pediatric patients has been studied in several studies, including premature infants (Potts et al., 2009; Su et al., 2016; Liu et al., 2017). However, although only one pharmacokinetic study of intranasal administered dexmedetomidine has been reported in children in cardiac surgery (Miller et al., 2018), no such data exist in Chinese children. Therefore, the aim of this study was to characterize the pharmacokinetics of intranasal administered dexmedetomidine in Chinese children. Based on previous pharmacokinetic data in adult and pediatric populations (Iirola et al., 2011; Li et al., 2018; Miller et al., 2018), we chose a dose of 1 µg/kg, expecting that this should provide adequate sedation and analgesia in the early postoperative phase.

## Materials and Methods

This prospective study in a single medical central was conducted in accordance with the guidelines for Good Clinical Practice and the Declaration of Helsinki. The study was approved by the Ethics Committee of the Second Affiliated Hospital of Wenzhou Medical University, China (Reference No. 2017-62) on July 25, 2017. The trial was registered at clinicaltrials.gov on November 9, 2017 (NCT03337581).

### Patients

The enrolment period was from October 20, 2017, to October 21, 2018. As this was not a confirmatory but an exploratory study, sample size estimation was not performed. Sixteen pediatric patients, aged 3 to 10 years, who were scheduled to undergo orthopedics, lower abdominal, urologic, or plastic surgery, were eligible for this study. The expected operation time was at least 2 h. Written informed consent was obtained from the parents or a legal guardian. Exclusion criteria included patients with a history of head trauma history, dexmedetomidine allergy, abnormal liver and kidney function, cardiac conduction system disease, current treatment with digoxin, alpha-adrenergic, or beta-adrenergic agonists or antagonists, clonidine, anti-arrhythmic medications, anticonvulsants, or presence of life-threatening medical conditions. Patients with acute nasal or respiratory symptoms on the day of the study were excluded because of potential interference with intranasal absorption.

### Clinical Protocol

Due to the duration of blood sampling, all participants were scheduled for anesthetization at 8:30 am. For safety, the patients were fasted from 2:30 am on the study day until 3 h after study drug administration. Intake of clear fluids was allowed until 5:30 am on the study day. There was no premedication used. Heart rate, non-invasive arterial blood pressure, peripheral arterial oxygen saturation, and ECG were monitored continuously. Anesthesia was induced with intravenous administration of fentanyl, 2 μg/kg and propofol, 2 to 3 mg/kg. Endotracheal intubation was facilitated with rocuronium 0.6 to 1.0 mg/kg before radial arterial cannulation. Mechanical ventilation was carried out with a mixture of 50% nitrous oxide and 50% oxygen. During surgical procedure, anesthesia was maintained with sevoflurane 2 to 3 vol%. Repetitive bolus doses of fentanyl 1 μg/kg were given if necessary. The attending anesthesiologist conducted the anesthesia to maintain heart rate and mean arterial blood pressure within 20% of baseline values.

### Dexmedetomidine Dosing

A dose of 1 µg/kg preservative-free dexmedetomidine (100 µg/ml, Jiangsu Hengrui Pharma Corporation, China) was prepared in a 1-ml syringe. Dexmedetomidine was not diluted and was stored at room temperature for 20 min to avoid irritating the nasal cavity of children. Thirty minutes before the end of surgery, the patient’s head was tilted back to fully expose the nasal cavity. The 1-ml syringe was used to enter the nasal cavity for about 0.5 cm, and the dose of 1 µg/kg dexmedetomidine was administered. After the administration, the nose was squeezed softly to promote the absorption of the drug solution, and the original body position was maintained for 2 min to prevent the drug solution flowing into the oral cavity through the nasal cavity.

### Blood Sampling and Drug Assay

Arterial blood samples of 0.5 ml each were collected at the following times: immediately before and 3, 5, 10, 15, 20, 30, 45, 60, 90, 120, 150, and 180 min after the intranasal administration. Due to clinical circumstances, longer arterial blood sampling was not feasible. Blood samples were preserved into ethylenediaminetetraacetic acid (EDTA) tubes and stored at 4°C. All heparinized blood samples were centrifuged within 30 min after collection, and the supernatant plasma was pipetted into glass vials and stored at −20°C immediately after separation. Later on, the samples were stored at −80°C until analysis.

Sample preparation was performed by liquid–liquid extraction. Plasma aliquots of 90 μl were mixed with 10 µl of internal standard solution and extracted with ethyl acetate (500 µl). After loading, the sample were vortexed about 2 min. Then, the sample was centrifuged at 15,000 rpm for 10 min, and the supernatant (450 μl) was transferred into 1.5-ml EDTA tubes. Extracts were evaporated to dryness with a gentle stream of nitrogen, and the dry residue was dissolved in 50-µl water. Finally, the sample was centrifuged at 15,000 rpm for 10 min, and the supernatant (40 μl) was transferred into EDTA tubes.

Isocratic ultraperformance liquid chromatography was performed with an ACQUITY UPLC BEH C18 1.7-µm 2.1 mm × 50 mm column (UPLC, Waters Corp., Milford, MA) and a mobile phase consisting of acetonitrile and 0.1% formic acid in water (28:72; flow rate 0.3 ml/min) at 37°C. A gradient program was used with the mobile phase combining solvent A (0.1% formic acid in water) and solvent B (acetonitrile) as follows: 0.0 to 0.5 min at 28% B, 0.5 to 1.5 min linear increase to 90% B, 1.5 to 2.0 min at 90% B, and 2.0 to 2.1 min at 28% B. A subsequent re-equilibration time was followed by 1 min of starting gradient condition. The flow rate was 0.30 ml/min, and the injection volume was 5 µl. The column and sample temperatures were maintained at 25°C and 4°C, respectively.

Mass spectrometric detection was carried out with an Applied Biosystems API 550 triple-quadrupole instrument, using positive ion spray ionization and multiple reaction monitoring. The needle potential was set to 1.3 kV, the source voltages cone was set to 35 V, the temperature of the heated purified air was 300°C, and the desolvation gas flow was set to 600 L/h. The collision energy was set to 20 V. The precursor ion–fragment ion pairs detected were m/z 200.99–94.98 for dexmedetomidine and m/z 205.039–99.014 for the internal standard d4-dexmedetomidine. Quantitation was based on peak area ratios of the analyte and the internal standard.

The assay was linear over a concentration range from 0.01 to 10.0 ng/ml with a correlation coefficient of 0.999. The within-run coefficient of variation was 12.2% at 0.01 ng/ml, 11.5% at 0.20 ng/ml, 4.75% at 5 ng/ml, and 0.58% at 10 ng/ml. The between-batch coefficient of variation was 1.34% at 0.20 ng/ml, 6.21% at 5 ng/ml, and 1% to 2% at 0.01 and 10 ng/ml. Mean recoveries of dexmedetomidine were better than 82.1%. The recovery of the internal standard (10 ng/ml) was 89.1 ± 4.6%. The matrix effect in children plasma was 83.5% to 103.2% for dexmedetomidine at different QC levels. The matrix effect for internal standard (10 ng/ml) was 92.2 ± 6.2%. No apparent matrix effect was found to affect the determination of dexmedetomidine and internal standard in children plasma.

### Pharmacokinetic Modeling

One-, two-, and three-compartment models with first-order absorption were evaluated for pharmacokinetic modeling of dexmedetomidine. The models were parameterized using apparent values of elimination clearance (CL/F), intercompartmental clearances (Q_2,3_/F), central volume of distribution (V_1_/F), peripheral volumes of distribution (V_2,3_/F), and the first-order absorption rate (K_a_). The analysis was performed with NONMEM (version 7.4.3, ICON Development Solutions), using the first-order conditional estimation method with interaction.

The model parameters were assumed to follow log-normal distributions and the interindividual variability for each structural parameter was modeled as:

*P* *_i_* = *P*_TV_ · exp (ခ*η* *_i_*) where P_i_ represents the parameter for the *i*th individual, P_TV_ is the typical value of the parameter in the population, and η_i_ is a random variable with zero mean and variance of ω^2^.

Residual variability was modeled using a combined proportional and additive error: *Cm* *_ij_* = *Cp* *_ij_* + *Cp* *_ij_* · ε_prop,_ *_ij_* + ε_add,_ *_ij_* where Cm*_ij_* is the *j*th observation of the *i*th subject, Cp*_ij_* is the corresponding predicted value, and ε_prop,_ *_ij_* and ε_add,_ *_ij_* are random variables with mean of zero and variance of σ^2^_prop_ and σ^2^_add_, respectively.

Model selection was based on the objective function value (OFV) using likelihood ratio tests and on residual analysis. A decrease in the OFV >6.63 (*P* = 0.01, χ^2^ distribution with one degree of freedom) was considered statistically significant.

Covariate analysis was conducted in two steps after the base model was identified. First, scatter plots of selected parameter estimates from the basic model versus potential covariates were plotted to explore the covariate-parameter relationships. In the second step, based on the identified potential covariates in step 1, covariate relationships with a linear or a power function were assessed for continuous covariates. The influence of body weight (BW) was modeled as ${\text{P}}_{i}={\text{P}}_{STD}\cdot {\left(\frac{BWi}{70}\right)}^{PBW}$ where P_STD_ is the standardized value of the parameter for a subject of 70 kg BW. For the power coefficient PBW, we tested a linear proportional model with PBW = 1 for all parameters, an allometric power model with PBW = 1 for volumes, and PBW = 0.75 for clearances, and a model where the values of PBW were estimated. Further potential covariates included sex, age, serum creatinine (Cr), the ratio of blood urea nitrogen to serum Cr (BUN/Cr), and Cr clearance (CrCL), which was calculated using the Cockcroft–Gault formula (Gault et al., 1992).

A stepwise approach was used to evaluate covariate effects. The selecting criteria for a covariate to be included in the model were based on a decrease of >3.84 in the OFV (*P* = 0.05, χ^2^ distribution with one degree of freedom) in the forward adding step and an increase of greater than 6.63 in the OFV (*P* = 0.01, χ^2^ distribution with one degree of freedom) in the backward deleting step.

Goodness of fit was assessed graphically and numerically. Model diagnostic graphs included: measured concentrations vs. population predictions and vs. individual predictions; ratio of measured to predicted concentration versus time; conditional weighted residuals versus time and versus population predictions.

Prediction errors were determined for individual and population predictions, and goodness of fit was assessed by the median prediction error (MDPE) and the median absolute prediction error (MDAPE) as follows:

$$\text{MDPE}=\text{median}\left(\frac{cm_{ij}-cp_{ij}}{cp_{ij}}\times 100\%\right)$$

$$\text{MDAPE}=\text{median}\left(\frac{cm_{ij}-cp_{ij}}{cp_{ij}}\times 100\%\right)$$

where Cm_ij_ and Cp_ij_ are the *i*th measured and predicted concentration of the *j*th individual, respectively.

A bootstrap analysis with 1,000 replicates (random resampling per subject, with replacement) was performed to analyze the stability of the model parameter estimates and to obtain nonparametric confidence intervals of the model parameters.

Prediction-corrected visual predictive check was used to graphically assess the appropriateness of the pharmacokinetic model (Bergstrand et al., 2011). For this purpose, the concentration profiles were simulated 1,000 times and compared with the observed data to evaluate the predictive performance of the model.

From the individual Bayesian estimates of the model parameters, the following pharmacokinetic variables were also determined: fast distribution half-life (T½α), terminal elimination half-life (T½β), and time to maximum plasma concentration after intranasal administration (Tpeak). The influence of BW was elucidated by simulating the pharmacokinetics for subjects of different BW.

### Statistics

Data are reported as mean ± standard deviation (SD) or median (range) unless stated otherwise. All model parameters are reported as estimated values with associated relative standard errors (RSE). Statistical analysis and simulations were performed using Matlab^®^ R2015b (MathWorks, Natick, MA) and R (version 3.5.1).

## Results

### Subject Characteristics

We totally recruited 16 patients. One patient withdrew during the trial and two patients were excluded due to blocked arterial catheterization. In five patients, it was not possible to collect all scheduled blood samples due to clinical circumstances. The last sample was drawn at 150 min in three subjects, at 120 min in one subject, and at 60 min in another subject. In the remaining eight subjects, the last sample was drawn at 180 min as scheduled. Therefore, the pharmacokinetic analysis was based on 145 samples from 13 children. Demographic and baseline clinical data are summarized in **Table 1**.

**Table 1:** Demographic and baseline clinical data, mean ± SD (range) or number.

Characteristics	Values
Sex (female/male)	8 / 5
Age (years)	6.5 ± 1.7 (4–10)
Weight (kg)	24.8 ± 5.8 (14–36)
Height (cm)	110 ± 12 (83–130)
Body mass index (kg/m^2^)	20.2 ± 1.0 (18.5–21.9)
Cr (µmol/L)	37.3 ± 7.3 (23.8–47.0)
BUN/Cr	0.11 ± 0.03 (0.04–0.15)
CrCL (mL/min)	99.2 ± 19.0 (78.2–150)

Cr, serum creatinine; BUN/CR, ratio of blood urea nitrogen and creatinine; CrCL, creatinine clearance.

### Pharmacokinetic Modeling

**Figure 1:**
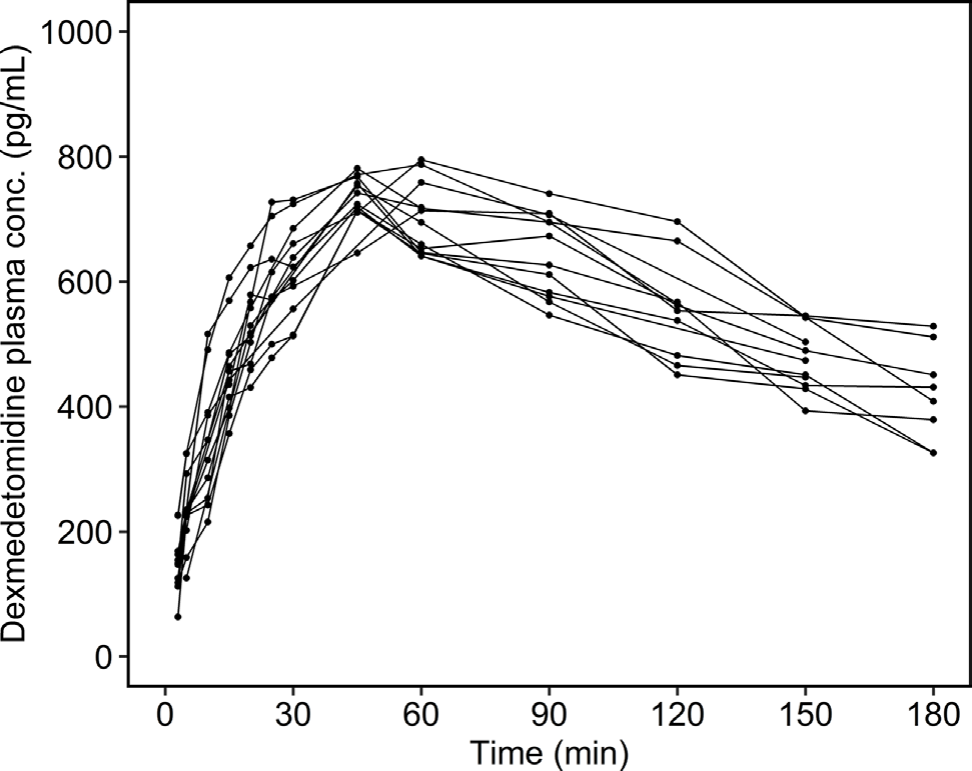
Measured dexmedetomidine plasma concentrations. Each line shows the data of one patient.

The individual time courses of the dexmedetomidine plasma concentrations are shown in **Figure 1**. There were no concentrations below the limit of quantitation. The maximum measured dexmedetomidine plasma concentration was 748 ± 29.6 (713–795) pg/ml, and the observed time to maximum concentration was 49.6 ± 7.21 (45.0–60.0) min.

A linear two-compartment model with first-order absorption (OFV = 1331.0) was significantly better than a one-compartment model (OFV = 1344.6), whereas a three-compartment model could not be estimated reliably. An additional lag time for first-order absorption did not further improve the fit (OFV = 1330.5). Therefore, a two-compartment model with first-order absorption was chosen as basic structural model. As the estimate of the proportional residual error σ^2^_prop_ was < 0.0001, this error term was removed from the model, keeping only the additive residual error σ^2^_add_. For the basic model, the ε shrinkage was 13.0%, and the η shrinkage of Ka, CL/F, V_1_/F, Q_2_/F and V_2_/F was 34.5%, 7.05%, 26.5%, 36.2%, and 18.1%, respectively. Scatter plots of the individual Bayesian parameters indicated an increase of CL/F, V_1_/F, and V_2_/F with weight. The influence of weight was best described by the allometric power model (OFV = 1287.1) with an exponent of 1 for all volumes and of 0.75 for all clearances. The linear weight model with an exponent of 1 for all parameters showed a worse fit (OFV = 1288.1). Estimation of the power exponents did not improve the model (OFV = 1286.6 with four additional parameters to be estimated). The interindividual variances of Ka, CL/F, V_1_/F, Q_2_/F, and V_2_/F changed from 17.3%, 27.0%, 28.9%, 112%, and 40.7% without weight effect to 10.6%, 14.5%, 7.55%, 162%, and 0.316% with allometric weight scaling. After inclusion of BW as covariate, there were no further significant effects of age, sex, and laboratory parameters detected.

The estimated parameters of the final pharmacokinetic model are summarized in **Table 2**. The interindividual variance of V_2_ was <0.0001 and was therefore fixed to zero. The fast distribution half-life in the population was 1.48 ± 1.45 (0.294–5.16) min, the terminal elimination half-life was 117 ± 16.1 (98.0–149) min, and the simulated time to maximum plasma concentration after intranasal administration was 55.2 ± 5.96 (42.3–63.4) min.

**Table 2:** Final population pharmacokinetic parameters.

	Estimate (RSE%)	Bootstrap median (95% CI)
Pharmacokinetic parameters		
Ka (1/min)	0.0378 (10.2%)	0.0379 (0.0315–0.0448)
CL/F (L/min/70 kg)	0.319 (6.6%)	0.318 (0.283–0.359)
V_1_/F (L/70 kg)	34.2 (14.4%)	33.5 (20.3–44.0)
Q_2_/F (L/min/70 kg)	10.0 (22.2%)	10.6 (5.11–34.1)
V_2_/F (L/70 kg)	34.9 (6.5%)	35.3 (29.4–46.1)
Interindividual variability ω^2^		
Ka	0.0112 (50.0%)	0.00949 (0.00107–0.0203)
CL /F	0.0207 (38.7%)	0.0178 (0.00452–0.0335)
V_1_ /F	0.00569 (82.7%)	0.00516 (0.00001–0.0174)
Q_2_ /F	1.29 (50.6%)	1.12 (0.203–3.18)
V_2_ /F	0 *(fixed)*	0 *(fixed)*
Residual variability		
σ^2^_add_	1752 (14.6%)	1742 (1285–2181)

Ka, absorption rate constant; CL/F, apparent clearance; V_1_/F, apparent central volume of distribution; Q_2_/F, apparent intercompartmental clearance; V_2_/F, apparent peripheral volume of distribution; RSE%, relative standard error; CI, confidence interval.

**Figure 2:**
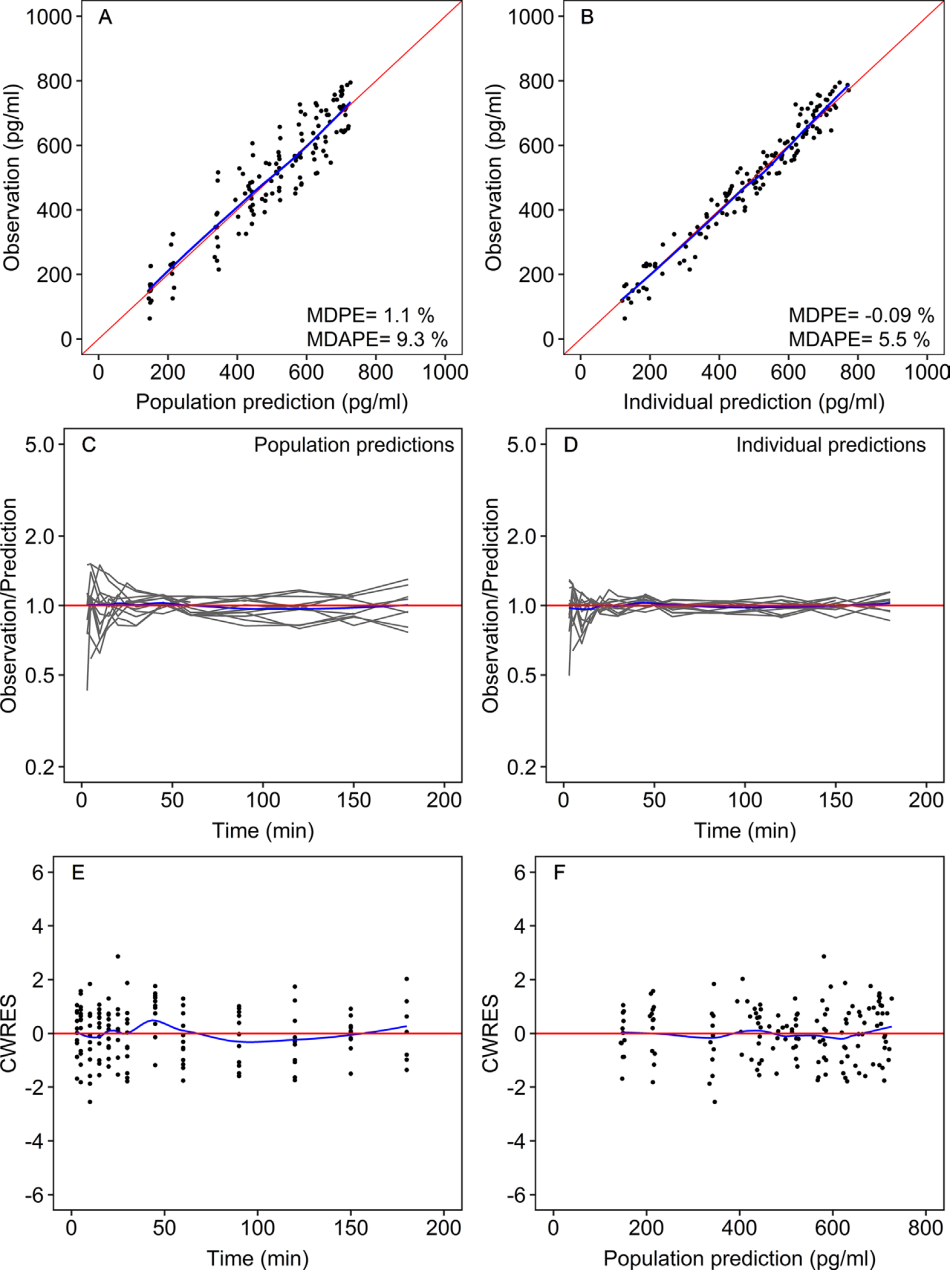
Goodness-of-fit plots for the final population model. Measured dexmedetomidine concentrations versus the population predictions **(A)** and versus the individual Bayesian predictions **(B)**. Ratio of the measured to predicted concentrations vs. time for the population predictions **(C)** and the individual predictions **(D)**. Conditional weighted residuals (CWRES) versus time **(E)** and versus population predicted plasma dexmedetomidine concentrations **(F)**. Each gray line shows the data of one patient. The red lines are lines of identity (measured = predicted). The blue lines are smoothers through the data. MDPE, median prediction error; MDAPE, median absolute prediction error.

**Figure 3:**
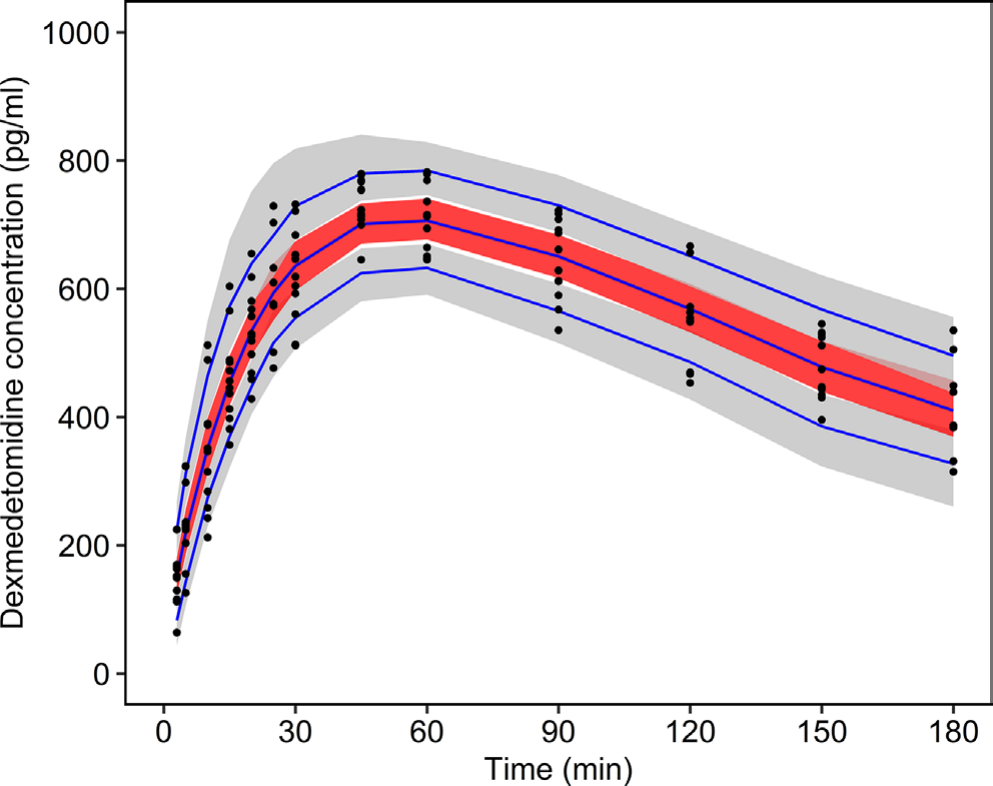
Prediction-corrected visual predictive check. The measured dexmedetomidine concentrations are plotted as black dots. The blue lines show the 5%, 50%, and 95% quantiles of the predictions. The gray shaded areas show the 90% confidence intervals of the 5% and 95% prediction quantiles. The red shaded area shows the 90% confidence interval of the 50% prediction quantile.

Median values and confidence intervals of the bootstrap estimates were in good agreement with the population estimates (**Table 2**). The goodness of fit plots for the final population pharmacokinetic model revealed a high quality of fit with small prediction errors and homogenously distributed residuals (**Figure 2**). The visual predictive check demonstrated the sufficiency of the model’s predictive power (**Figure 3**).

**Table 3** shows the simulated effects of BW on the pharmacokinetics of dexmedetomidine after intranasal administration. Due to the allometric weight scaling, the half-lives are not constant but increase with BW. With increasing BW, the maximum plasma concentration is expected to occur later and to be higher.

**Table 3:** Pharmacokinetic parameters for a child of 10, 20, 30, and 40 kg body weight, as obtained from the final pharmacokinetic model.

	10 kg	20 kg	30 kg	40 kg
CL/F (L/min)	0.0740	0.124	0.169	0.209
V_1_/F (L)	4.88	9.76	14.6	19.5
V_SS_/F (L)	9.87	19.7	29.6	39.5
T½α (min)	0.73	0.87	0.96	1.03
T½β (min)	93.1	111	123	132
T_peak_ (min)	52.4	55.6	57.5	58.9
C_peak_ (pg/ml)	675	704	720	731

CL/F, apparent clearance; V_1_/F, apparent central volume of distribution; V_SS_/F, apparent volume of distribution at steady-state; T½*α*, fast distribution half-life; T½*β*, terminal elimination half-life; T_peak_, time to maximum plasma concentration after intranasal bolus administration; C_peak_, maximum plasma concentration after an intranasal bolus dose of 1 µg/kg.

### Side Effects

**Figure 4:**
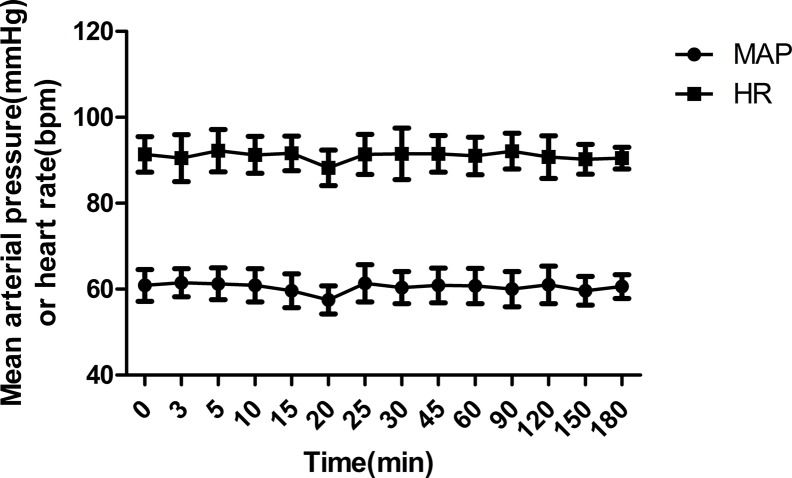
Mean arterial blood pressure (MAP) and heart rate (HR) during the study period (mean ± SD).

Dexmedetomidine slightly decreased the blood pressure and heart rate after intranasal administration (**Figure 4**). When compared with the baseline, a maximum decrease of 5.4 ± 4.7% for mean arterial blood pressure and 3.4 ± 0.9% for heart rate was observed 20 min after administration of dexmedetomidine. The administered dose of 1 µg/kg provided sufficient sedation in the early postoperative phase. There were no cases of postoperative emergence delirium. The wake-up time after discontinuation of sevoflurane was 20.3 ± 3.8 min, and the retention time from the postanesthesia care unit was 32.7 ± 7.1 min.

## Discussion

It was the aim of this study to characterize the pharmacokinetics of intranasal dexmedetomidine in Chinese children. A two-compartment model with first-order absorption and allometric BW scaling was found to describe the data appropriately, whereas age, sex, and laboratory parameters did not show any effect on weight normalized pharmacokinetic parameters.

There are currently only three studies on the pharmacokinetics of intranasal dexmedetomidine available, of which two are in adults (Yoo et al., 2015; Li et al., 2018) and only one in children (Miller et al., 2018). Yoo et al. (2015) administered 84 µg (approximately 1 µg/kg) nasal dexmedetomidine to six healthy adults and found that the peak arterial plasma concentration was 340 (230–700) pg/ml, and the peak time was 38 (15–60) min. Li et al. (2018)reported for adults a median peak concentration of 250 and 280 pg/ml after 1 µg/kg dexmedetomidine administered by an atomizer or as drops, respectively. In children aged 6 to 48 months, Miller et al. (2018) found a peak concentration of 182 (163–251) pg/ml and a peak time of 47 (31–62) min after 1 µg/kg dexmedetomidine. The peak time of 45 to 60 min in the present study is in good agreement with Miller’s findings, whereas the shorter peak time in the study by Yoo et al. might have been caused not only by the different age but also by the usage of a higher concentrated dexmedetomidine formulation (500 µg/ml vs. 100 µg/ml). However, the peak concentration after 1 µg/kg dexmedetomidine in the present study was 713 to 795 pg/ml, which is about three times higher than that in the other studies on intranasal dexmedetomidine. Concerning the study by Miller, one has, however, to consider the younger age in that study (6–48 months vs. 4–10 years in the present study). There may be also differences in bioavailability, which has been reported to vary between 41% (Li et al., 2018) and 84% (Miller et al., 2018). Finally, dexmedetomidine was applied as drops in the present study, whereas it was administered by an atomizer in the study by Miller et al. (2018). Although Li et al. (2018) did not find differences between application by drops or by atomizer in adults, one cannot rule out such an influence of the intranasal administration in young children. The circumstance that the head was tilted back with the children in a supine position might also have influenced the absorption of dexmedetomidine in the present study. Another possible reason for the observed differences might be alterations in pharmacokinetics due to ethnicity, as the present study was performed in Chinese children whereas Miller et al. and Yoo et al. studied Caucasians. Li et al. found that maximum plasma concentrations after 1 μg/kg in healthy Chinese adults were similar as those reported for Caucasian children and adults in studies by Miller and Yoo. However, in a previous study with intravenous administration of dexmedetomidine, Liu et al. (2017) found that Chinese children showed a similar clearance, but larger volumes of distribution when compared with studies in Caucasians. Altogether, such pharmacokinetic alterations due to ethnicity alone may not fully explain the higher concentrations observed in the present study.

Corresponding to the higher observed concentrations, the present estimates of clearance and volume of distribution were lower than in previous studies. Whereas the apparent elimination clearance was 0.32 L/min per 70 kg in the present study, Miller et al. found a systemic clearance of 1.04 L/min per 70 kg with a bioavailability of 84%. The apparent central volume of distribution in the present study was 34.2 L/70 kg compared with 59.8 L/70 kg in the study by Miller. However, one has to consider the differences in age and in ethnicity between the two studies. Furthermore, Miller et al. studied cardiac patients, whereas we studied children undergoing orthopedics, lower abdominal, urologic, or plastic surgery. In a previous study in Chinese children aged 1 to 9 years, Liu et al. (2017) found a systemic clearance of 0.60 L/min/70 kg and a central volume of distribution of 84.3 L/70 kg after short intravenous infusion, which is also higher than in the present study. However, one has to consider that venous blood samples were analyzed in that study, whereas arterial concentrations were used in the present study.

Concerning the effect of covariates on dexmedetomidine pharmacokinetics, previous studies found a significant influence of BW (Potts et al., 2009; Liu et al., 2017; Miller et al., 2018). In the present study, BW was also found to be a significant covariate, and as in previous studies, an allometric scaling with an exponent of 0.75 for clearances and an exponent of 1.0 for volumes was most appropriate to assess this influence. The estimation of the power exponents did not improve the model. Furthermore, Anderson and Holford (2008) proposed to fix the allometric coefficients because estimates of these parameters may be quite imprecise. A model with a linear proportional weight scaling with an exponent of 1.0 for all parameters was only slightly worse than the allometric model. This may be explained by the relatively narrow range of BW (14–36 kg) in the present study, so that the difference between a power model with an exponent of 0.75 and a linear proportional model is not as pronounced. On the other hand, the allometric scaling of clearance with an exponent of 0.75 is an established and well-supported model to account for the effect of size in pharmacokinetics (Anderson and Holford, 2008). Whereas the interindividual variabilities of the apparent elimination clearance CL/F and the apparent volumes of distribution were clearly reduced after BW scaling, this was not the case for the intercompartmental clearance Q2/F. This means that the large interindividual variability of this parameter remains unexplained. One has, however, to consider that Q2/F was the parameter with the highest shrinkage and the highest RSE. Additionally, the standard error of the interindividual variability for this parameter was also relatively high. Thus, this parameter was not as well estimable as the other parameters. This might be caused by the relative small sample size of 13 subjects and the relatively short sampling time of 180 min. The power model for the weight effect has the consequence that the maximum plasma concentration after intranasal administration should occur later and should be higher for a child with higher BW (**Table 3**). Furthermore, the recovery might be longer as the terminal half-life also increases with weight. However, these weight effects are only small or moderate.

Age had no effect on dexmedetomidine pharmacokinetics in the present study. This is reasonable if one considers that the age effect on clearance, which was reported by Potts et al. (2009), was most prominent in the younger than 2 years range, whereas the age in our study population was 4 to 10 years. The present finding that renal function as assessed by CrCL had no effect is also in agreement with previous findings (De Wolf et al., 2001).

In previous studies, it was also found that dexmedetomidine clearance was affected by the cardiac output in a way that dexmedetomidine reduces cardiac output, which in turn leads to a reduction of dexmedetomidine clearance (Dutta et al., 2000; Iirola et al., 2012; Li et al., 2018). In this study, cardiac output was not monitored. However, the reported cardiac output effect was prominent at dexmedetomidine concentrations greater than 1,000 pg/ml, which were not reached in this study. As the observed changes in blood pressure and heart rate were also small, one may assume no effect of dexmedetomidine on the cardiac output in the present study.

The effect of dexmedetomidine on hemodynamics is mainly dependent on plasma drug concentration and intravenous injection rate, and a biphasic cardiovascular response has been reported for dexmedetomidine with a transient hypertension followed by hypotension (Ebert et al., 2000). The transient hypertension can be avoided by dosing regimens with a slow increase of concentration (Dyck et al., 1993). Dexmedetomidine causes peripheral vasoconstriction and bradycardia when the plasma concentration exceeds 1,000 pg/ml (Ebert et al., 2000). In the present study, mean arterial pressure and heart rate slightly decreased after 20 min of administration, but no increase in blood pressure was observed. This may be explained by the slow concentration increase after nasal administration compared with intravenous administration and by the relatively low peak plasma concentrations.

Concerning the therapeutic window of dexmedetomidine for sedation in children, it has been proposed that a range of 200 to 600 pg/ml may be appropriate for procedural sedation (Miller et al., 2018). The plasma concentrations in the present study were higher, but the sedation in the early postoperative phase was satisfactory without serious side effects. One may therefore conclude that an intranasal dose of 1 µg/kg is appropriate for sedation in the early postoperative phase in Chinese children in the studied range of age and weight.

The present study has several limitations. First, the blood sampling time was only 3 h, whereas a longer sampling time might have allowed the estimation of a three-compartment model. In previous pharmacokinetic studies of intravenous and also intranasal administration of dexmedetomidine in children, however, a two-compartment model was also found to be appropriate, even if longer blood sampling was performed (Potts et al., 2009; Liu et al., 2017; Miller et al., 2018). A sampling time of about three times the terminal half-life (i.e., 6 h for dexmedetomidine) is generally recommended for pharmacokinetic studies. Due to clinical circumstances it was, however, not feasible to collect arterial blood samples longer than 3 h, and a combination of venous and arterial samples would have also not been appropriate. Furthermore, the terminal half-life of about 2 h in the present study is in agreement with the findings of previous studies. In five subjects, the last concentration was drawn before 180 min. The impact on the results, however, was negligible, as the parameter estimates were very similar when these five subjects were removed from the data set. A further limitation is the circumstance that there was no intravenous administration of dexmedetomidine performed, so that the bioavailability after intranasal administration could not be determined. Finally, as the range of age and weight in the study population was relatively small, we cannot draw conclusions about the pharmacokinetics of intranasal administered dexmedetomidine in infants younger than 4 years or in obese children.

In conclusion, the pharmacokinetics of intranasal dexmedetomidine could be well described by a two-compartment model with first-order absorption and allometric BW scaling. When compared with the studies in Caucasians, Chinese children showed a similar time to peak plasma concentration, but the achieved plasma concentrations were about three times higher. The reason for this observation is not completely clear, but differences in age, ethnicity, and mode of administration may be responsible. Therefore, further studies in larger populations would be helpful.

## Data Availability

Publicly available datasets were analyzed in this study. This data can be found here: www.clinicaltrials.gov.on

## Author Contributions

Z-YH and JC contributed to the drug assay. X-FY, FC and YL contributed to the sample collection. JS contributed to the pharmacokinetic analysis. C-YW and HI contributed to the pharmacokinetic analysis and writing of the article. Q-QL and H-CL contributed to the study design.

## Funding

The research was funded by Zhejiang provincial natural science foundation (LY17H310006), Clinical Research Foundation of the Second Affiliated Hospital of Wenzhou Medical University (SAHoWMUCR2018-03-126) and Zhejiang provincial public welfare technology application research foundation of China (2015C33100).

## Conflict of Interest Statement

The authors declare that the research was conducted in the absence of any commercial or financial relationships that could be construed as a potential conflict of interest.
